# Is breastfeeding really *invisible*, or did the health care system just choose not to notice it?

**DOI:** 10.1186/1746-4358-3-13

**Published:** 2008-08-04

**Authors:** Chris Mulford

**Affiliations:** 1 WIC Breastfeeding Initiative, Southern New Jersey Perinatal Cooperative, USA

## Abstract

There are innumerable myths and misconceptions about breastfeeding that minimize its importance; these often keep health workers from providing effective care to support and protect breastfeeding. They are compounded by lack of basic and applied research, and by the cultural invisibility of breastfeeding in the United States. This paper highlights some of the blind spots and suggests the importance of an approach that places breastfeeding promotion and advocacy within the context of women's lives. As we work to ensure that the health care system provides good breastfeeding care, we need to guard against letting the medicalization of infant feeding keep us from remembering that breastfeeding is something that mothers and children do, in all the aspects of their private and public lives.

## Debate

As a nurse and lactation consultant working for three decades in maternity care and with WIC (the Special Supplemental Nutrition Program for Women, Infants, and Children), I have encountered innumerable myths and misconceptions about breastfeeding, in addition to attitudes that so minimize its importance that it just gets overlooked. These "blind spots" often keep health workers from providing effective care to support and protect breastfeeding. They are compounded by lack of basic and applied research, and by the cultural invisibility of breastfeeding in the United States.

Although a "breastfeeding renaissance" began with La Leche League in the 1950s, much of the basic research into breast function and normal infant feeding, behavior, and growth has only recently been done. Much remains undone. In 2005, Ramsay and her colleagues published information from ultrasound studies of the lactating breast [1]. They updated the classic images of the mammary duct system that were based on dissections done over 150 years before – in 1840 [1]. Many other questions, such as determining the normal ranges for feeding frequency and output of newborns in the first few days after birth are still unanswered.

Even when research is undertaken, it is not always clear that it is addressing the right questions. Brown and colleagues investigated US$40 million in federal funds that were allocated for research on infant nutrition, breastfeeding, and lactation from 1994–1996 [2]. In 1990, the nation set goals to increase initiation and duration of breastfeeding, but less than 14% of the funding actually went to projects that measured initiation and duration of breastfeeding as outcome variables; 7.5% of the studies, reflecting $4.1 million, were designed to investigate ways to improve artificial milk or develop new pharmaceuticals and therapies [2].

Breastfeeding is practiced by relatively vulnerable people (mothers and babies/young children). Almost no one makes money from it. Almost no one advertises it. In everyday life you almost never see it or hear people talk about it. Most U.S. babies breastfeed under cover. Mothers breastfeed toddlers and pre-schoolers "in the closet." The functional (lactating) breast is viewed as the "abnormal" breast, and lactation as a temporary aberration. The fragmentation of reproductive health care, which divides responsibility for breastfeeding among several specialties, was a recipe for having *nobody *care much about or take responsibility for breastfeeding ... until 1985, when the lactation consultants came along. Lactation consultants make breastfeeding their central focus, but they are still fighting to win recognition and respect as a profession.

**Figure 1:**
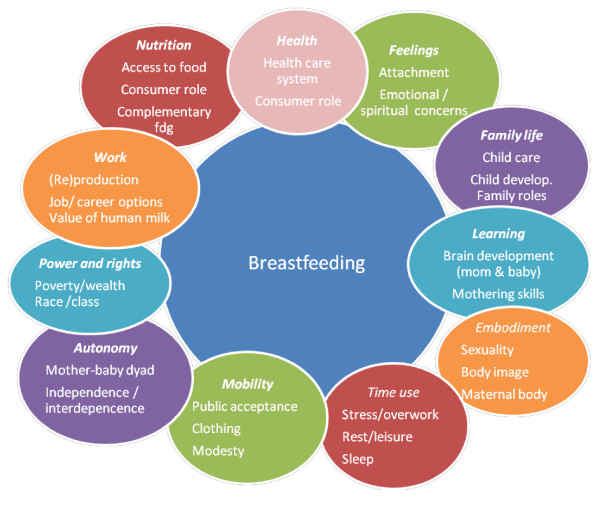
**Breastfeeding in context**. It is important to be aware that the behavior of breastfeeding can affect and be affected by all of a woman's roles. Breastfeeding is more than a way to provide nutrition, more than a health choice, more than a method of care.

Yet while we are thinking about problems in the health care system, we should remember that breastfeeding is only partly a "health care" issue. "Breastfeeding in context" (Figure 1) is an attempt to show breastfeeding in the setting of a woman's life, surrounded by her roles and activities, like force fields that affect and are affected by breastfeeding. It is important that we work to ensure that the health care system provides good breastfeeding care, but we shouldn't let the medicalization of infant feeding keep us from remembering that breastfeeding is something that mothers and children *do*, in all the aspects of their private and public lives.

I will end with a partial list of blind spots in medical/nutritional/clinical knowledge/attitudes about breastfeeding/lactation.

• We assume that breastfeeding is "natural," therefore nobody needs to be taught about it. At the same time, we assume that breastfeeding is difficult, therefore only well-educated women with access to resources can do it; and that breastfeeding can only be taught by someone who has done it herself.

• We assume that breastfeeding is a personal decision; therefore women who do not plan to do it do not need to know anything about it.

• We assume that men do not need to know anything about infant feeding either.

• Since lactation is a temporary, short-term physiological function we assume that the "normal" breast is the non-lactating breast and, therefore, we do not have to develop techniques for assessing lactating breasts but only need wait for the woman to stop breastfeeding... or tell her to stop if she needs a mammogram or other diagnostic test.

• Breast surgery techniques attempt to improve breast appearance often to the detriment of breast function.

• Reliable and valid methods of estimating the adequacy of milk intake by breastfeeding babies are still being refined. For years we have been only guessing about how much "pee" and "poo" babies should put out and how much weight they should lose or gain in the first week.

• The normal values for infant blood glucose in the first 48 hours after birth are still a subject of debate.

• We do not know why women get engorged when their milk "comes in," and we are still debating how to treat engorgement, which is a major and painful event for many mothers.

• It took a decade to collect data for global infant growth charts based on exclusively breastfed babies, and now it is in doubt whether U.S. doctors will even agree to use them.

• The recommended dietary allowance (RDA) of calories for babies based on theoretical research was set too high in the 1970s and 1980s. This led to the false belief that breastfed babies required supplementary foods at an early age. Research on actual breastfed babies showed that they grow just fine on much less intake than called for by the standard recommendation. [3] However, even when the experts realized the standard was set too high and should have been lower, they did not re-set it at a realistic level because they said it was more "prudent ... when in doubt to err on the side of caution." [4]

• Drugs are not tested on lactating women, so there is *no *information from drug companies on drug safety for this important population. Information is gathered in dribs and drabs, by studying the rare women who value breastfeeding enough to find a doctor to help them assess their babies' reaction and safety while they take the drugs in question, and then report the findings. We also need more choices of contraceptive methods that are safe and effective to use during lactation.

## Competing interests

The author declares that she has no competing interests.
